# Inducible and coupled expression of the polyomavirus middle T antigen and Cre recombinase in transgenic mice: an *in vivo* model for synthetic viability in mammary tumour progression

**DOI:** 10.1186/bcr3603

**Published:** 2014-01-23

**Authors:** Trisha Rao, Jill J Ranger, Harvey W Smith, Sonya H Lam, Lewis Chodosh, William J Muller

**Affiliations:** 1 Rosalind and Morris Goodman Cancer Research Centre, McGill University, Montreal, QC, Canada; 2 Ontario Cancer Institute, University Health Network, Toronto, ON, Canada; 3 Abramson Family Cancer Research Institute, University of Pennsylvania, Philadelphia, PA, USA

## Abstract

**Introduction:**

Effective *in vivo* models of breast cancer are crucial for studying the development and progression of the disease in humans. We sought to engineer a novel mouse model of polyomavirus middle T antigen (PyV mT)-mediated mammary tumourigenesis in which inducible expression of this well-characterized viral oncoprotein is coupled to Cre recombinase (TetO-PyV mT-IRES-Cre recombinase or MIC).

**Methods:**

MIC mice were crossed to the mouse mammary tumour virus (MMTV)-reverse tetracycline transactivator (rtTA) strain to generate cohorts of virgin females carrying one or both transgenes. Experimental (rtTA/MIC) and control (rtTA or MIC) animals were administered 2 mg/mL doxycycline beginning as early as eight weeks of age and monitored for mammary tumour formation, in parallel with un-induced controls of the same genotypes.

**Results:**

Of the rtTA/MIC virgin females studied, 90% developed mammary tumour with complete penetrance to all glands in response to doxycycline and a T_50_ of seven days post-induction, while induced or un-induced controls remained tumour-free after one year of induction. Histological analyses of rtTA/MIC mammary glands and tumour revealed that lesions followed the canonical stepwise progression of PyV mT tumourigenesis, from hyperplasia to mammary intraepithelial neoplasia/adenoma, carcinoma, and invasive carcinoma that metastasizes to the lung; at each of these stages expression of PyV mT and Cre recombinase transgenes was confirmed. Withdrawal of doxycycline from rtTA/MIC mice with end-stage mammary tumours led to rapid regression, yet animals eventually developed PyV mT-expressing and -non-expressing recurrent masses with varied tumour histopathologies.

**Conclusions:**

We have successfully created a temporally regulated mouse model of PyV mT-mediated mammary tumourigenesis that can be used to study Cre recombinase-mediated genetic changes simultaneously. While maintaining all of the hallmark features of the well-established constitutive MMTV-PyV mT model, the utility of this strain derives from the linking of PyV mT and Cre recombinase transgenes; mammary epithelial cells are thereby forced to couple PyV mT expression with conditional ablation of a given gene. This transgenic mouse model will be an important research tool for identifying synthetic viable genetic events that enable PyV mT tumours to evolve in the absence of a key signaling pathway.

## Introduction

Since its discovery in the 1950s, studies on the polyomavirus middle T antigen (PyV mT) have been essential in understanding cellular signalling and tumourigenesis. PyV mT can readily transform cells *in vitro* and *in vivo* by stimulating key pro-tumourigenic signalling axes. Phosphorylation of the membrane-anchored PyV mT protein by Src family tyrosine kinases leads to the recruitment and activation of specific proto-oncogenes such as Shc and phosphatidyl inositol 3’ kinase. In recent decades, the signalling pathways initiated by these effectors have been found to be activated in several human tumours and, consequently, PyV mT model systems continue to be employed in cancer research (reviewed extensively in [1]).

Transgenic expression of PyV mT in the mouse can lead to tumour formation in many epithelial tissues [2]. Perhaps most valuable to the field of breast cancer is the widely used mouse mammary tumour virus (MMTV)-driven PyV mT mouse model, in which transgene expression occurs in the mammary epithelium [3]. Mammary tumour development in this strain closely mimics the disease progression observed in humans, evolving through four distinct stages: hyperplasia, mammary intraepithelial neoplasia (MIN)/adenoma, early carcinoma and late carcinoma [4]. Another clinically relevant feature of this model is that PyV mT-induced mammary tumours are capable of metastasizing to the lungs [3]. First published two decades ago, the MMTV-PyV mT strain has since become an established tool in studying breast tumourigenesis and metastasis *in vivo*.

Initial attempts to study the mammary-specific role of a particular gene in the MMTV-PyV mT model involved crossing in LOXP-flanked alleles of this gene as well as an MMTV-driven Cre recombinase transgene. However, the MMTV promoter is only active in approximately 70% of the mammary epithelium, resulting in stochastic Cre recombinase expression in PyV mT tumour tissue; a selection for PyV mT-positive/Cre recombinase-negative cells arises in the developing tumour, precluding excision of the conditional allele of interest. This phenomenon has occurred in several MMTV-PyV mT/MMTV-Cre recombinase backgrounds as demonstrated by our own laboratory: conditional ablation of β1-integrin, focal adhesion kinase, c-Src or Stat3 was prevented, indicating that in each case the conditional gene was most likely essential to PyV mT-driven tumourigenesis [5-7] (unpublished observations; Ranger JJ, Muller WJ). The idea of coupling Cre recombinase expression to that of the oncogene to circumvent this selection process was successfully demonstrated in the MMTV-NIC (Neu-IRES-Cre recombinase) model of ErbB2/Neu breast cancer, in which a mutant Neu transgene is linked to Cre recombinase through an internal ribosome entry sequence (IRES) [8]. Every mammary tumour cell expresses both Neu and Cre recombinase and, thus, any conditional loci present in the genome of this cell should be recombined and completely ablated.

In addition to replicating this coupling strategy for a new PyV mT strain, we sought to expand the applicability of the model to temporal regulation. The recent departure from constitutive or hormone-responsive promoters in transgenic breast cancer mouse models (for example, MMTV) to a chemically-inducible approach has been made possible by the advent of the MMTV-reverse tetracycline transactivator (rtTA) strain used in combination with the well-established TetON system [9]. The tetracycline-inducible promoter is only turned on in response to the tetracycline derivative, doxycycline, in contrast to the hormone-responsive MMTV promoter that becomes constitutively active at approximately three weeks of age. By turning on expression of a tetracycline-responsive transgene in the adult mouse, one can avoid potential complications caused by overexpression of the oncogene or by Cre recombinase-mediated removal of a LOXP-flanked cassette during development; likewise, expression can then be turned off after tumour formation to investigate the possibility of regression and recurrence. It should be noted that a TetON-PyV mT mouse strain has been reported which is sensitive to inducible mammary tumour progression in the presence of the MMTV-rtTA transgene; however, PyV mT is not coupled to Cre recombinase in this case [10].

In order to link expression of the PyV mT oncogene with that of Cre recombinase in an inducible manner, we generated a TetO-PyV mT-IRES-Cre recombinase (MIC) transgenic mouse that, when crossed to the MMTV-rtTA strain and treated with doxycycline, expresses both PyV mT and Cre recombinase from the same bi-cistronic transcript in the mammary epithelium. In the majority of experimental mice, mammary tumours develop within two weeks of induction, progress through the typical PyV mT histological stages, and metastasize to the lung. These tumours were susceptible to regression upon doxycycline withdrawal; however, recurrent tumours ultimately arose in de-induced animals. This research article details the characterization of this novel inducible model and reflects on its potential use in future studies of PyV mT mammary tumourigenesis.

## Methods

### Generation of the MIC construct and rtTA/MIC bigenic strain

The MIC construct was created using the pTE-mElf5-IRES-eGFP vector (a generous gift from Dr. C. Ormandy). Briefly, after removal of the mElf5 and eGFP transgenes, PyV mT cDNA was sub-cloned between the Tet-operator (TetO) and internal ribosome entry sequence (IRES), followed by sub-cloning of Cre recombinase cDNA downstream of the IRES (TetO-PyV mT-IRES-Cre recombinase; abbreviated as MIC) (Additional file 1: Figure S1). Derivation of the MIC strain was conducted in the Transgenic Core Facility in the Goodman Cancer Research Centre using standard pronuclear injection of FVB/N single cell embryos [11]. Progeny were screened for germ-line transmission of the MIC transgene by PCR genotyping. MMTV-reverse tetracycline transactivator (rtTA) transgenic mice were generated in the laboratory of Dr. Lewis Chodosh as previously described [9]. The ROSA26 Cre recombinase-activated β-galactosidase reporter strain (GTRosa) was generated in the laboratory of Dr. Phillipe Soriano as previously described [12]. All mice were housed in the animal facility at the Goodman Cancer Research Centre. Ethical approval was obtained for the use of animals and all experiments were done in accordance with the animal care guidelines at the Animal Resource Centre of McGill University. All transgenic animals described in this study were on a pure FVB/N background.

### Doxycycline induction of rtTA/MIC mice and necropsy procedures

Experimental and control animals of at least eight weeks of age were given drinking water containing 2 mg/mL doxycycline (Sigma Aldrich, St. Louis, MO, USA) in light-blocking bottles each week. Mammary tumours were detected by physical palpation and animals were sacrificed at a total tumour burden of six cubic centimetres unless otherwise specified. Material from necropsied mice was flash frozen in liquid nitrogen (in some cases, tissues were set in an OCT medium before freezing) or fixed in 10% neutral buffered formalin (Leica Microsystems Inc., Concord, ON, Canada) and embedded in paraffin wax. Fixed and embedded mammary tissues were sectioned at 4 μm and either stained by haematoxylin and eosin (H&E) or processed further as indicated. H&E-stained lung sections (4 μm) were scanned with a Scanscope XT Digital Slide Scanner and for each sample the area occupied by metastases relative to the total lung area was quantified using Imagescope software (Aperio, Vista, CA, USA). The number and type of lesions in mammary gland sections were counted similarly by analysing 10 fields within a scanned image of each sample. Inguinal mammary glands were whole mounted on glass slides and processed for H&E-staining as described [6]. For resection surgeries, mice were anesthetised prior to excising a small piece of tumour for embedding and flash freezing; the incision was sutured and painkillers administered for one week.

### Immunoblotting

Flash frozen mammary tissues were lysed as in [6] and 20 μg of protein were separated by SDS-PAGE. Membranes were immunoblotted with the following primary antibodies: E-cadherin (BD Biosciences, Mississauga, ON, Canada; 610182, 1:1,000); Cre recombinase (Novagen/EMD Millipore, Billerica, MA, USA; 69050, 1:1,000); P-EGFR (Y1068) (Cell Signaling/New England Biolabs, Pickering, ON, Canada; #3777, 1:1,000); EGFR (Cell Signaling #2322, 1:1,000); P-ErbB2 (Y1248) (Santa Cruz Biotechnology, Santa Cruz, CA, USA; #12352, 1:500); ErbB2 (Santa Cruz Biotechnology #284, 1:1,000); Hsp90 (Cell Signaling #4874); PyV mT (a generous gift from Dr. S. Dilworth, Ab750, 1:1000), c-Myc (Santa Cruz Biotechnology #764); P-PDGFRβ (Y1021) (Cell Signaling #2227, 1:1,000); PDGFRβ (Cell Signaling #3175, 1:1,000); α-tubulin (Cell Signaling #2125). Horse radish peroxidase (HRP)-conjugated secondary antibodies were purchased from Jackson ImmunoResearch Laboratories, West Grove, PA, USA. GE Amersham (Bai d'Urfe, QC, Canada) enhanced chemiluminescence detection reagents were used to visualize the immunoblots.

### Immunohistochemistry

Sections of paraffin-embedded samples were immunostained as described previously [6]. Antigen retrieval was performed in 10 mM sodium citrate buffer. Sections were blocked in a solution of Power Block Universal Blocking Agent (Biogenex , Fremont, CA, USA) and incubated with primary antibodies: PyV mT (a generous gift from Dr. S. Dilworth, Ab762, 1:100) or Cre recombinase (Covance, Denver, PA, USA; PRB 106C, 1:600). In the case of PyV mT, the sections were incubated with anti-mouse secondary antibody conjugated to HRP (Dako, Burlington, ON, Canada; #K4006). For Cre recombinase, the sections were incubated first with biotinylated anti-rabbit secondary antibody (Jackson ImmunoResearch Laboratories) and then with avidin conjugated to HRP (Vector Labs, Burlington, ON, Canada; Vectastain Elite ABC kit #PK-6100). Development was carried out by exposing with DAB reagent (Dako #K3467). Slides were counterstained with 20% haematoxylin before mounting.

### β-galactosidase assay

OCT-embedded sections of mammary tumours were processed and stained with X-gal as described previously [6].

### qRT-PCR

Total RNA was extracted from flash frozen mammary tumours and lung lesions using a Qiagen AllPrep DNA/RNA Mini Kit (Qiagen Inc., Toronto, ON, Canada; #80204). cDNA was prepared by reverse transcribing the isolated RNA using M-Mulv Reverse Transcriptase (#M0253S), Oligo-dT(23VN) (#S1327S) and a murine RNase inhibitor (#M0314S) (all purchased from New England Biolabs). Real-time quantitative PCR was performed on the cDNA using LightCycler 480 SYBR Green I Master (Roche, Missisauga, ON, Canada; #04707516001) and run on a Roche LightCycler 480 instrument. Primers used for PyV mT were as follows: forward - 5’CCCGATGACAGCATATCCCC-3’; reverse - 5’CTTGTTCCCCCGGTAGGATC-3’. PyV mT transcript levels were normalized to GAPDH transcript levels.

### DNA sequencing

Total DNA was extracted from flash frozen mammary tumours and lung lesions using a Qiagen AllPrep DNA/RNA Mini Kit (#80204). Genomic DNA was analysed by Sanger sequencing (Applied Biosystems 3730 DNA Analyser; Life Technologies Inc., Burlington, ON, Canada) using the following primers to detect SNPs in known mutational "hotspots" of *Hras*, *Kras1*, *Nras* and *Trp53*:

*Hras* exon2/exon3

Forward - CCTTGGGTCAGGCATCTATT

Reverse - AAAGACATAAAGCCTCAGTGTGC

*Kras* exon 2

Forward- GAAGATGAAAGTACTGGTTTCCA

Reverse- TGCACCTATGGTTCCCTAACA

*Kras* exon 3

Forward - TCACCTTGTAAAAGATGCACTG

Reverse - AAAACAGGAATTCTGCATACTTGA

*Nras* exon 2

Forward - AGTGGAAGGCCACGTGTATC

Reverse - GGAAATCCTCAGTAAGCACGA

*Nras* exon 3

Forward - TGCATGCGTGTGATTATGTATG

Reverse - AAAAGTTGTATGTTTCCTAAGTCCA

*Trp53* exon 2

Forward - ACGTGGTTGGTTACCTCTGC

Reverse - GATACAGGTATGGCGGGATG

*Trp53* exons 3/4

Forward - CCAGCCTGGGATAAGTGAGA

Reverse - GCTAAAAAGGTTCAGGGCAAA

*Trp53* exons 5/6

Forward - TGGTGCTTGGACAATGTGTT

Reverse - CCCTTCTCCCAGAGACTGCT

*Trp53* exons 7/8/9

Forward - GTAGGGAGCGACTTCACCTG

Reverse - AAGACCTGGCAACCTGCTAA

*Trp53* exon 10

Forward - GTTGGGAACCAACTTTCAGA

Reverse - TGTCCCTCATACCCCTTAACA

*Trp53* exon 11

Forward - CAGAAGTATTCCAGTGTGTTCTGTG

Reverse - CTACTCAGAGAGGGGGCTGA

### Phospho-RTK array

The Proteome Profiler Mouse Phospho-receptor tyrosine kinase (RTK) Array Kit from R&D Systems/Cedarlane Corp., Burlington, ON, Canada (#ARY014) was used according to the manufacturer’s instructions. Flash frozen mammary tissues were lysed as in [6] and 200 μg was used for each array. Arrays spotted with RTK probes were incubated with biotin-conjugated phospho-tyrosine antibody (EMD Millipore #16-103, 1:1,000) followed by fluorescently-conjugated streptavidin (Mandel Scientific, Guelph, ON, Canada; #LIC-926-32230, 1:1,000) to allow for visualization on the Odyssey Imaging System (LI-COR Biosciences, Lincoln, NE, USA). Fluorescence intensities for each probe were normalized to a PBS probe (negative control).

## Results

### Induction of MIC transgene expression in the mammary gland results in rapid tumour onset

Polyomavirus middle T antigen (PyV mT) and Cre recombinase cDNAs were sub-cloned into a pTE vector containing an internal ribosome entry sequence (IRES) to produce a TetO-PyV MIC transgene (Additional file 1: Figure S1). MIC virgin females were aged to one year without issue and the transgene did not disrupt normal breeding in either sex or nursing by females. MIC founder lines were crossed to the MMTV-rtTA strain to drive doxycycline-inducible transgene expression to the mammary epithelium [9]. Tumour onset in the original constitutive MMTV-PyV mT model occurs with relatively short onset, with virgin females developing mammary masses with a T_50_ of 40 days of age [3,6]. To evaluate whether this was also the case for the MIC model, we induced cohorts of rtTA/MIC, rtTA and MIC virgin female mice between 8 and 16 weeks of age with 2 mg/mL doxycycline which has been previously shown to lead to robust expression of the Tet-inducible transgene in an MMTV-rtTA background [9]. The minimum age of eight weeks for induction was chosen to ensure that the mammary epithelium would be almost fully developed. Induced animals were initially examined every other day by physical palpation for mammary tumour formation, alongside un-induced controls of the same genotypes. A single founder line in which mammary tumours were detected was chosen for further breeding to generate cohorts that would be more extensively characterized.

**Figure 1:**
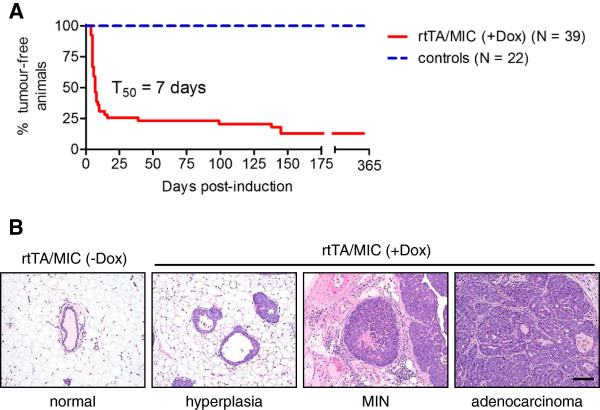
**Induced rtTA/MIC animals develop mammary tumours with characteristic histopathological features of PyV mT-driven mammary tumourigenesis. (A)** Kaplan-Meier mammary tumour onset curve as measured by physical palpation. A total of 87.1% (34/39) of mice developed multifocal mammary tumours with a T_50_ of 7 days and an average of 22.0 ± 7.1 days post-induction with doxycycline. Control animals (induced rtTA or MIC; un-induced rtTA/MIC) were monitored for at least one year. **(B)** Representative H&E-stained sections of normal ductal structures in a mammary gland from a control animal (an un-induced rtTA/MIC) followed by typical stages of PyV mT mammary tumour progression (hyperplasia, mammary intraepithelial neoplasia (MIN), and adenocarcinoma) in mammary glands and tumours from rtTA/MIC mice following doxycycline induction. (Scale bar: 100 μm).

Mammary gland masses were detected in rtTA/MIC mice as early as four days post-induction, with 74.4% (29/39) of the cohort developing multifocal tumours within 16 days of induction (Figure 1A). The few animals that did not palpate within this short 16-day window of induction could be subdivided into two groups: those that developed tumours between 17 and 365 days post-induction (12.8%; 5/39) and those that remained tumour-free after one year of induction (12.8%; 5/39). Considering the entire rtTA/MIC induction cohort, the average tumour onset was 22.0 ± 7.1 days post-induction while the T_50_ was 7 days of induction, reflecting the very rapid and complete induction observed in the majority of animals. Precise regulation of the MIC transgene was evident based on the concurrent observations that rtTA/MIC mice developed mammary tumours exclusively and that all control animals (both induced and un-induced) remained tumour-free after one year post-induction.

Tumour growth in rtTA/MIC mice progressed differently from what has been observed in the MMTV-PyV mT model. In the latter, tumours develop as focal masses in each gland that are easily measurable. At defined time-points, histological analysis of the inguinal mammary glands from MMTV-PyV mT mice shows a gradient of transformation, with the older and more advanced lesions proximal to the nipple, and newer lesions at earlier stages of tumourigenesis towards the terminal end buds of the epithelial network [13]. While distinct masses are initially palpable in an induced rtTA/MIC mouse, the entire gland promptly thickens within days, making it difficult to perform calliper measurements at this stage. Animals sacrificed at onset (approximately four days post-induction) or two weeks post-induction harboured inguinal mammary glands filled with early lesions (data not shown; Additional file 2: Figure S2). This difference between the two models could be explained by the fact that constitutive PyV mT-mediated transformation occurs during puberty as the ductal epithelial network progressively penetrates the fat pad, while in the MIC model transformation was initiated in an almost mature gland.

All tumour-bearing rtTA/MIC females were sacrificed at a total tumour volume of approximately six cubic centimetres (denoted as “end-stage”). Histological analysis of mammary glands and tumours from these animals revealed the presence of all previously characterized stages of PyV mT tumourigenesis, ranging from hyperplasia, to MIN/adenoma, and finally to early and late carcinoma (Figure 1B; Additional file 3: Figure S3B). Adjacent mammary gland whole mounts from tumour-bearing mice were also fully transformed (Additional file 3: Figure S3A). Mammary gland sections and whole mounts from age-matched control animals were normal (Figure 1B; Additional file 4: Figure S4). It appeared that our novel inducible PyV mT strain was closely recapitulating the histological stepwise tumour progression documented in the MMTV-PyV mT model.

### rtTA/MIC mammary tumours co-express the PyV mT oncogene and a functional Cre recombinase

**Figure 2:**
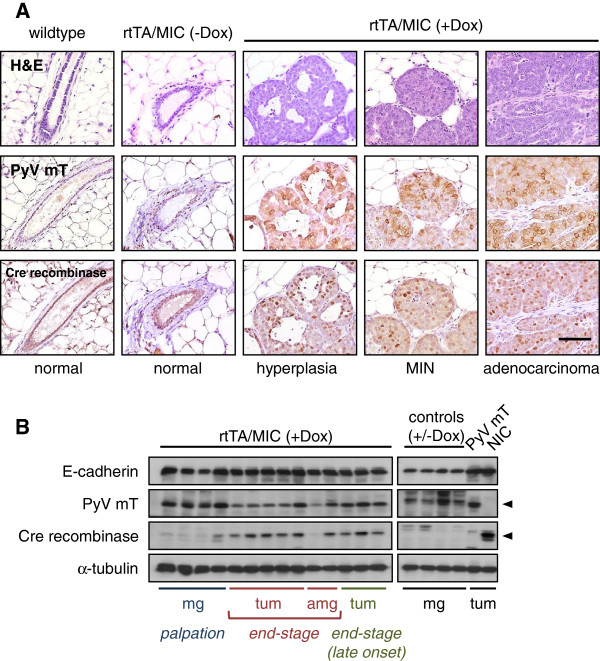
**PyV mT and Cre recombinase are expressed at all stages of tumourigenesis. (A)** Immunohistochemical detection of PyV mT (middle row) and Cre recombinase (bottom row) in ductal structures at the indicated stages of mammary tumour progression in rtTA/MIC mice, contrasted by normal mammary gland histology from control (an un-induced rtTA/MIC) and wildtype mice. The corresponding H&E-stained sections are shown for comparison (top row). (Scale bar: 100 μm). **(B)** Immunoblot analysis of protein lysates from rtTA/MIC mammary glands (mg), mammary tumours (tum) and adjacent mammary glands (amg) from animals sacrificed at palpation or at end-stage (“late onset” refers to palpation after 16 days of induction) as indicated using antibodies directed to E-cadherin (epithelial content control), PyV mT, Cre recombinase and α-tubulin (loading control). Controls include (from left to right) mammary glands from two un-induced rtTA/MIC mice, one induced MIC mouse and one induced rtTA mouse. Positive controls for PyV mT and Cre recombinase expression were mammary tumours from MMTV-PyV mT (PyV mT) and MMTV-NIC (NIC) animals, respectively; arrowheads indicate specific bands for these proteins.

Having established that mammary tumours were indeed inducible in the rtTA/MIC system, our next step was to verify expression of the MIC transgene by immunohistochemistry. PyV mT and Cre recombinase antibodies stained the membrane and nuclei, respectively, of cells in rtTA/MIC lesions in a mosaic pattern (Figure 2A). Notably, normal ductal epithelium in both age-matched controls and wildtype animals did not stain positively for PyV mT or Cre recombinase.

To confirm MIC transgene expression by immunoblot, protein extracts were prepared from mammary glands and tumours from rtTA/MIC mice sacrificed at palpation or at end-stage tumour burden (“late onset” refers to palpation after 16 days of induction). These lysates were positive for PyV mT expression; Cre recombinase was also detected in rtTA/MIC tumours, although it was lowly expressed in mammary gland lysates which likely have relatively less epithelial content as indicated by E-cadherin levels (Figure 2B). Induced and un-induced control mammary glands did not express PyV mT or Cre recombinase protein.

**Figure 3:**
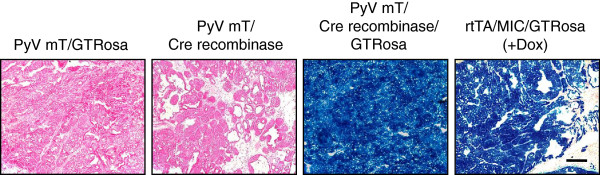
**Cre recombinase expression and activity are uniform in the epithelium of rtTA/MIC mammary tumours.** X-gal staining (blue) of mammary tumour sections from rtTA/MIC animals carrying the Cre recombinase-activated β-galactosidase reporter transgene (GTRosa). Negative controls include MMTV-PyV mT mammary tumours with either GTRosa (PyV mT/GTRosa) or MMTV-Cre recombinase transgenes (PyV mT/Cre recombinase). An MMTV-PyV mT mammary tumour with both transgenes (PyV mT/Cre recombinase/GTRosa) was used as a positive control. Samples were counterstained with nuclear fast red (pink). (Scale bar: 500 μm).

In order to use this model for Cre recombinase/LOXP-mediated excision of genes, we needed to confirm that the Cre recombinase produced from the MIC transgene was functionally active. To accomplish this, we utilized a Rosa26-β-galactosidase reporter strain (“GTRosa”) in which the *lacZ* gene is downstream of a LOXP-flanked STOP cassette [12]. The presence of Cre recombinase allows for expression of the β-galactosidase enzyme from the *lacZ* transgene, which can cleave the compound X-gal into an insoluble blue-coloured product. The mammary epithelium of rtTA/MIC/GTRosa tumour sections turned blue upon staining with X-gal, indicating that Cre recombinase had been expressed and active in these cells (Figure 3). This outcome is comparable to an MMTV-PyV mT/MMTV-Cre recombinase/GTRosa mammary tumour in which there are no conditional alleles present. Collectively, these results demonstrate that the rtTA/MIC mouse model can be used to study Cre recombinase-dependent genetic alterations in conjunction with PyV mT oncogenic activation.

### The rapid induction of rtTA/MIC lesions is associated with metastatic dissemination of tumour cells to the lung

**Figure 4:**
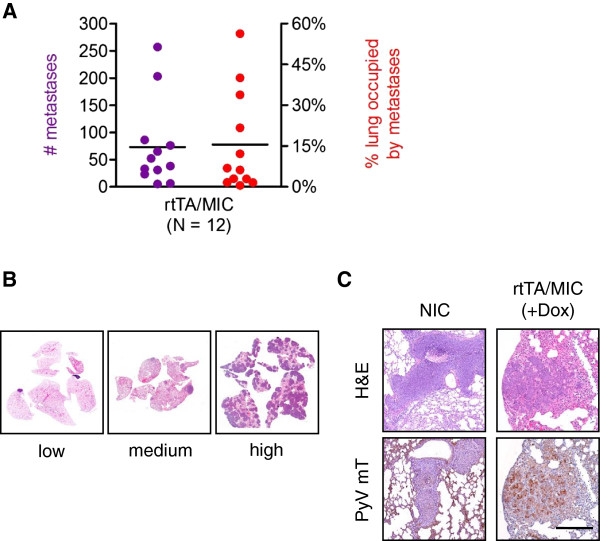
**rtTA/MIC end-stage mammary tumours exhibit a high capacity for metastatic dissemination to the lungs. (A)** Quantification of the number of metastatic lung lesions (left axis) and the percentage of lung tissue occupied by metastases (right axis) in rtTA/MIC animals at end-stage tumour burden. Bars represent the average value for each parameter. **(B)** H&E-stained whole lung sections representative of low, medium and high levels of metastasis. **(C)** Representative lung lesion from a tumour-bearing rtTA/MIC animal stained with H&E (top row) and PyV mT (bottom row). An MMTV-NIC (NIC) lung lesion is shown as a negative control for PyV mT staining (Scale bar: 200 μm).

One of the most useful features of the original constitutive PyV mT model is the ability of the mammary tumours to effectively metastasize to the lungs, an organ that is a common site of distal lesions in the human disease. To determine if rtTA/MIC mammary tumours were capable of forming pulmonary metastases, we examined sections of the lung lobes from animals that had reached similar end-stage tumour burdens. All tumour-bearing mice presented with lung metastases, albeit to varying degrees, with some lungs harbouring only a few small lesions while others were made up almost entirely of secondary tumour tissue (Figure 4A, B). These lung lesions stained positively for PyV mT, confirming that they derived from the primary rtTA/MIC mammary tumour (Figure 4C). Interestingly, there was no correlation between the extent of metastasis and tumour burden, extending the idea that PyV mT tumours are heterogeneous in their transforming capabilities and may thus also be in terms of malignancy (data not shown; [13]). Taken together, these observations demonstrate that this inducible MIC model reproduces many of the pathological features of the original MMTV-PyV mT strain.

### De-induction of the MIC transgene results in immediate tumour regression and eventual recurrence of doxycycline-independent masses

**Figure 5:**
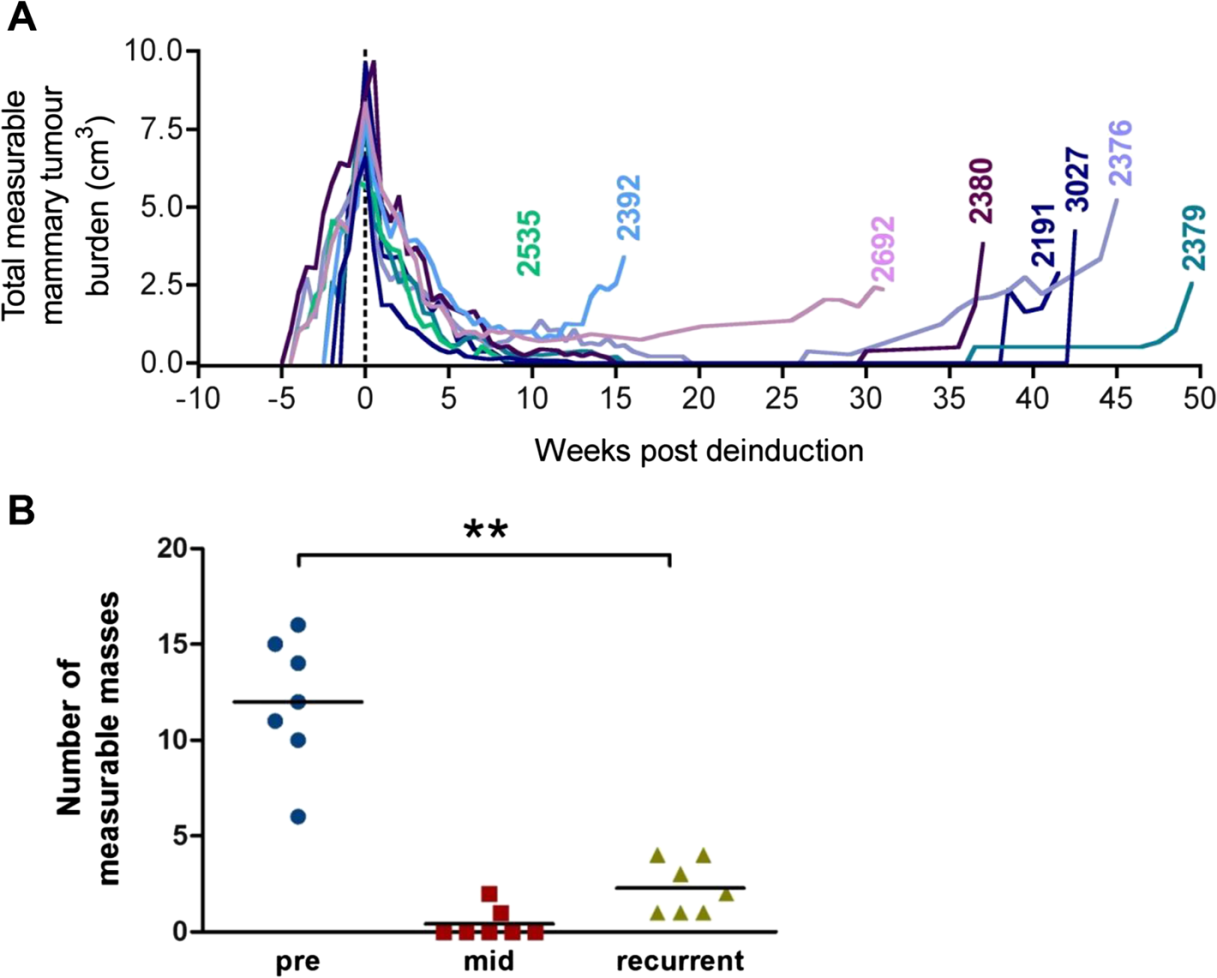
**Doxycycline withdrawal in rtTA/MIC tumours leads to rapid regression and eventual spontaneous recurrence of masses. (A)** Total mammary tumour burden measured over time in rtTA/MIC mice prior to and following doxycycline withdrawal (indicated by the dotted line at time 0). Tumour-bearing mice were de-induced upon reaching burden endpoint. Each line represents an individual animal labeled by its ID number. **(B)** Quantification of the number of measurable masses detected pre-regression (pre), at the point of maximum regression (mid) and at sacrifice (recurrent). Bars represent the average value at each time-point. **P <0.01.

Another important feature of inducible systems is the capacity to “turn off” the oncogene by withdrawal of the inducing agent; one can then evaluate whether tumours regress and if they have the potential to recur in the absence of transgene expression. To test this in our model, doxycycline treatment was discontinued for a cohort of rtTA/MIC mice bearing end-stage mammary tumours. Upon de-induction of PyV mT expression the tumours began to shrink rapidly (Figure 5A). By 10 weeks post-de-induction, most of the tumours had regressed to palpable masses that were no longer measurable. Interestingly, all of the de-induced rtTA/MIC mice eventually developed recurrent masses (15 to 50 weeks post-de-induction) and were sacrificed at burden endpoint. The number of measurable recurrent tumours arising was significantly less than the number of measurable masses the animal had prior to de-induction (Figure 5B). This suggests that the emergence of more focal, doxycycline-independent tumours in post-regression mice is a spontaneous event, in contrast to the consistent and complete penetrance of multifocal, doxycycline-dependent tumours driven by the inducible MIC transgene to all mammary glands of a given animal. Doxycycline-independent tumours arose only in rtTA/MIC mice and not in control animals de-induced at the same time, which remained normal (data not shown). Analysis of sections from mid-regression tumours and completely regressed mammary glands revealed relatively normal ductal structures surrounded by extensive stromal deposition and occasionally abnormal adipose tissue, which may explain why these glands remained palpable so long after de-induction (data not shown).

**Figure 6:**
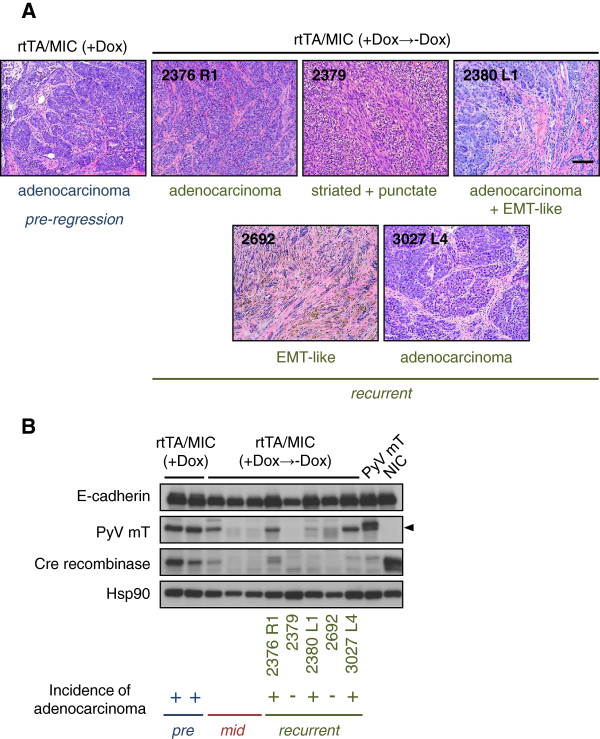
**Recurrent masses from de-induced rtTA/MIC mice have variable histopathologies and some exhibit re-expression of PyV mT. (A)** H&E-stained recurrent mammary tumours arising in rtTA/MIC mice post-doxycycline withdrawal. The mouse ID number (followed by the tumour location in the case of multiple recurrences, for example, “R1”) and histopathology of the tumour are indicated for each image. A pre-regression rtTA/MIC doxycycline-dependent mammary tumour exhibiting typical end-stage adenocarcinoma is shown for comparison. (Scale bar: 100 μm). **(B)** Immunoblot analysis of protein lysates from rtTA/MIC mammary tumours prior to and following doxycycline withdrawal using antibodies directed to E-cadherin (epithelial content control), PyV mT, Cre recombinase and Hsp90 (loading control); the arrowhead indicates the specific band for PyV mT protein. Resected mammary tumours were used for pre- and mid-regression time-points (pre- and mid-, respectively), while recurrent masses were harvested from mice sacrificed at clinical endpoint. The mouse ID numbers of the recurrent tumour lysates are indicated and correspond to the images labeled in **(A)**. The incidence of adenocarcinoma in the corresponding histological section for each sample is indicated by a “+” symbol.

The morphology of the recurrent masses exhibited striking intra- and intertumoural heterogeneity, with individual tumours differing among animals and among tumours from the same mouse (Figure 6A; Additional file 5: Figure S5A). Some histopathologies resembled pre-regression adenocarcinoma while others were more divergent, such as epithelial-mesenchymal-transition (EMT)-like and striated/punctate morphologies. All mice with recurrent tumours had evidence of lung metastases at sacrifice, varying in number, size and stage (data not shown). A single animal sacrificed prior to recurrent tumour development did not present with any metastases which suggests that, at least in this individual case, MIC-induced lung lesions may regress in parallel with the primary tumour upon doxycycline withdrawal.

To determine if the recurrent tumours were no longer dependent on the PyV mT oncogene, we subjected lysates from the five tumours shown in Figure 6A to immunoblot analysis (Figure 6B). Two samples clearly showed detectable bands at the expected size for PyV mT (2376 R1 and 3027 L4), while weaker signals were observed in two other samples (2380 L1 and 2692); one sample appeared to be completely negative for PyV mT expression (2379). To achieve a more quantitative assessment of PyV mT levels, qRT-PCR was carried out on these and other recurrent tumours as well as corresponding metastatic lung lesions from two animals. We found that PyV mT transcript levels in the recurrent tumours reflected the protein levels obtained by immunoblotting (Additional file 5: Figure S5B). For the most part, re-expression of the PyV mT transgene correlated with the incidence of adenocarcinoma in the tumour’s corresponding histological section. The lack of complete correlation can be explained by the fact that we must use different pieces of a histologically heterogeneous tumour for different analyses. Interestingly, a PyV mT transcript was detected in a metastatic lung lesion in addition to a recurrent tumour from the same animal (2376; Additional file 5: Figure S5B); PyV mT protein expression in the lung lesions was confirmed by immunohistochemistry (data not shown). On the other hand, in an animal with a PyV mT-expressing recurrent tumour (2380 L1) and a -non-expressing recurrent tumour (2380 L3), PyV mT transcript was undetectable in a metastatic lung lesion. This may reflect the presence of separate recurrent lesions that either re-expressed the transgene or arose in the absence of PyV mT transcript. It may additionally indicate the capability of non-PyV mT-expressing, recurrent mammary lesions to metastasize to the lungs, or the PyV mT-independent recurrence of an originally doxycycline-dependent lung metastasis *in situ*. We should note that the lower transcript levels of PyV mT transgene in the rtTA/MIC tumours relative to that of the MMTV-PyV mT tumour are likely due to the different strengths of the promoters driving the transcription of the oncogene (TetON versus MMTV). This does not impact on overexpression of PyV mT protein in the inducible system, as evidenced by the strong levels detected by immunoblot (Figure 2B) and ultimately the fact that transformation occurs rapidly in the model.

It appeared that while some recurrent tumours arose by re-expressing the PyV mT transgene, others did so by alternative mechanisms. In an attempt to identify these potential mediators of recurrence, we used an antibody array to analyse phospho-RTK levels in primary and recurrent tumour lysates and focused on candidates with relatively high fluorescence intensities, specifically epidermal growth factor receptor (EGFR), ErbB2 and platelet-derived growth factor receptor (PDGFR) β (Additional file 6: Figure S6A). While not all candidates validated by immunoblotting, we did observe phosphorylation of both ErbB2 and PDGFRβ in at least some of the recurrent tumours, suggesting that RTK signalling was actively occurring (Additional file 6: Figure S6B). ErbB2 is known to be up-regulated during mammary tumour progression in the MMTV-PyV mT model [4]. While we observed relatively low expression of ErbB2 in our samples as compared to an MMTV-NIC control lysate, the strong levels of phosphorylated ErbB2 in the doxycycline-independent rtTA/MIC recurrences may represent an avenue of recapitulating the signalling associated with PyV mT tumourigenesis in the absence of transgene re-expression. This may be the case for 2379, which also showed overexpression of c-Myc protein; interestingly, amplification of the *c-Myc* gene has been observed in a model of recurrence after de-induction of the doxycycline-dependent oncogene [14].

To further analyze the potential occurrence of cooperating oncogenic events during the process of doxycycline-independent recurrence of rtTA/MIC mammary tumours, we sequenced regions of the three Ras genes (*Hras*, *Kras1* and *Nras*) and of *Trp53* that are orthologous to those frequently mutated in human cancers. Notably, mutations in these genes have been previously identified as potential driving events in the recurrence of other doxycycline-driven transgenic mouse tumour models [15,16]. No mutations were found in any of the genes examined in doxycycline-dependent rtTA/MIC mammary tumours (data not shown). In recurrent mammary tumours, we found no mutations in exons 2 and 3 (containing codons 12 and 61) of either of the Ras genes but did identify an arginine-to-cysteine mutation at residue 245 of *Trp53* (R245C) in one recurrent mammary tumour (2380 L1; data not shown). The affected residue corresponds to R248 of human *TP53*, which is frequently mutated in human cancer. This result suggests that mutations in known tumour suppressor genes can occur in recurrent rtTA/MIC mammary tumours. However, at least in the case of *Trp53*, they may be relatively infrequent (1/10 samples examined). A more comprehensive mutational analysis (for example, using exome sequencing) of doxycycline-dependent and recurrent rtTA/MIC mammary tumours could be undertaken in the future to provide additional information on cooperating genetic events during tumour recurrence.

Collectively, these data illustrate that, while we can demonstrate rapid tumour regression in rtTA/MIC animals by withdrawal of doxycycline, the emergence of doxycycline-independent tumours ultimately transpires. This can be attributed in at least some cases to the reactivation of the PyV mT transgene and corresponds with an adenocarcinoma phenotype. In other cases, tumour recurrence may be associated with activation of RTK signalling and/or cooperating oncogenic mutations, such as the observed mutation in *Trp53*. These events may correlate with a different spectrum of tumour histopathologies, since the occurrence of the R245C mutation in 2380 L1 correlates with the appearance of an EMT-like morphology in addition to adenocarcinoma (Figure 6A). This is in keeping with the established tendency of *Trp53* mutations to induce tumours with EMT-type histopathological features in transgenic mouse models [17].

## Discussion

The development of inducible transgene systems for *in vivo* studies has made it possible to more accurately model human diseases. The ability to control transgene expression in mice allows the researcher to initiate tissue-specific changes at relevant time-points and, in the case of oncogenic transgenes such as PyV mT, mimic disease initiation (induction) and treatment (de-induction). The TetO-PyV mT-IRES-Cre recombinase (MIC) strain generated in our laboratory not only utilizes inducible expression of the PyV mT oncoprotein, but incorporates Cre recombinase-mediated genetic changes as well, due to the bi-cistronic linking of these transgenes. In this study, we have chosen a mammary epithelial-specific rtTA (MMTV-rtTA) to characterize a new model of mammary tumourigenesis driven by the MIC transgene.

Induction of rtTA/MIC mice with doxycycline led to the rapid onset of invasive mammary tumours in the majority of animals. MIC-expressing lesions developed in a stepwise fashion that resembled the progression observed in the constitutive MMTV-driven model of PyV mT tumourigenesis (MMTV-PyV mT). Notably, earlier lesions displayed more uniform staining of PyV mT and Cre recombinase protein and at a higher intensity than carcinoma stages, suggesting a potential dampening of transgene expression once the tumour has progressed (Figure 2A). There may be a reduced requirement for strong PyV mT levels in an advanced lesion due to the presence of other genetic aberrations. Despite a rapid tumour onset, it may be possible for cooperating genetic or epigenetic events to occur in this short time period; in fact, constitutive expression of PyV mT was shown to be insufficient for transformation without additional changes in endogenous genes [13]. This hypothesis may be extended to the expression of Cre recombinase. The homogenous staining of rtTA/MIC mammary tumours obtained in the X-gal assay contrasts with the heterogeneous Cre recombinase expression determined by immunohistochemistry, which may simply be due to inherent differences between the two analyses (Figure 2A; Figure 3). We suspect that all of the mammary epithelial cells in these animals expressed Cre recombinase at some point at least once given that 100% of the tumour epithelium is positive for the β-galactosidase reporter; however, as Cre recombinase is only required for the initial recombination event, its expression may have been turned off during tumour progression. The functionality and tight regulation of Cre recombinase by doxycycline in this model was ultimately validated by our preliminary findings that conditional loss of the p110α isoform of phosphatidyl inositol 3’ kinase - a critical mediator of PyV mT signaling *in vitro* and hypothetically essential for tumourigenesis *in vivo* - is achievable in the rtTA/MIC model, leading to abrogation of tumour formation in animals induced for at least 200 days [18] (unpublished observations; Rao T, Muller WJ, Zhao JJ).

One important difference between the inducible PyV mT model and the conventional MMTV-PyV mT model is that tumour development in our inducible PyV mT model was not 100% penetrant. A proportion of the animals in our cohort either developed masses much later than 16 weeks post-induction or had no palpable masses prior to one year post-induction (Figure 1A). Despite the longer latency, the tumours from the late onset group resembled early onset tumours in terms of progression, metastatic capacity and MIC transgene expression (data not shown; Figure 2B). The delayed onset in some rtTA/MIC mice contrasts with the complete penetrance observed in both the MMTV-PyV mT strain and the previously published inducible PyV mT strain (TetO-PyV mT-IRES-Luciferase) [3,10]. An initial explanation would be that the discrepancy is due to differences in the number and/or location of the transgene integration site(s), especially given that specific loci appear to be more permissible than others. Another possible explanation is that the rtTA is non-responsive in certain transgene carriers. However, this is not likely the case, as demonstrated by the robust induction of hyperplastic lesions in mammary gland whole mounts from 100% (19/19) of animals following two weeks of doxycycline administration (Additional file 2: Figure S2A). Analysis of mammary gland sections at this time-point revealed that the ducts were almost exclusively abnormal (either hyperplastic or filled), in contrast to only normal ducts in a control animal (Additional file 2: Figure S2B, C). These data argue that the observed incomplete tumour penetrance is not due to technical issues with the inducible MIC system but rather reflects the natural history of tumour development and progression in this model. An additional theory for the difference in tumour penetrance deals with the fact that PyV mT and Cre recombinase are expressed during adulthood and may be subject to immune rejection, in contrast to the pre-pubescent onset of PyV mT expression in the conventional MMTV-PyV mT model. We may have some evidence to support this hypothesis: unpublished experiments in our laboratory have found that MMTV-NIC mammary tumour cells do not grow in immune-competent mice, while they readily propagate in immune-compromised hosts (for example, NCr, SCIDbeige) or in a tolerant environment, such as the MMTV-Cre recombinase strain. Moreover, MMTV-NDL mammary tumour cells, which lack Cre recombinase but express the same ErbB2/Neu oncogene used in the MMTV-NIC model, are tolerated in an FVB/N background (unpublished observations; Muller WJ). Future studies investigating the sustainability of rtTA/MIC mammary epithelial cells in immune-deficient mice should allow this issue to be addressed.

The inducibility of the MIC transgene and mammary tumour progression in our model was found to be reversible upon withdrawal of doxycycline; however, tumour recurrence became a reproducible phenomenon in de-induced animals (Figure 5). It is possible that the accruement of additional genetic lesions during doxycycline-dependent tumour progression allowed the regressed mammary glands to reinitiate tumourigenesis. Unlike typical end-stage rtTA/MIC mammary tumours, the recurrent masses were often focal and the remarkable array of histopathologies documented was unexpected, particularly considering the homogeneous nature of the initial doxycycline-dependent tumours which all presented as adenocarcinoma at end-stage (Figure 6A; Additional file 5: Figure S5A). As revealed by immunoblotting and qRT-PCR analyses of recurrent tumour lysates, reactivation of signalling may be attributable to re-expression of the MIC transgene in some cases (Figure 6B; Additional file 5: Figure S5B). Indeed, reactivation of inducible transgenes as a consequence of mutations in the rtTA has been reported previously and may present a possible explanation for a subset of our recurrent tumours [19]. From a clinical standpoint, the fact that tumours can evolve in the absence of PyV mT reactivation recapitulates the well-established problem of therapeutic resistance in breast cancer. The PyV mT oncogene is widely regarded as an RTK mimic and so the recurrence experiment could be thought of as another way of modelling recurrence of RTK-driven breast cancer upon chronic administration of an RTK inhibitor. Indeed, several of the recurrent tumours (including a non-PyV mT-expressing sample) showed expression and/or activation of known proto-oncogenes (Additional file 6: Figure S6), while one tumour also carried an oncogenic mutation in the *Trp53* tumour suppressor. In the latter case, we cannot exclude the possibility that the primary tumours that gave rise to these recurrences included tumour cell populations that had already undergone this genetic event. However, its absence in the panel of doxycycline-dependent samples we analysed suggests that it may also occur *de novo* during the process of tumour regression and recurrence. In summary, our rtTA/MIC model of de-induction appears to be a valuable system for investigating clinical treatment and relapse.

## Conclusions

We have demonstrated that our novel MIC mouse strain in conjunction with the MMTV-rtTA is a suitable model of PyV mT mammary tumourigenesis with the added benefit of temporal regulation. Our rtTA/MIC mice can be induced to develop metastatic tumours in a classical stepwise fashion, closely recapitulating the human disease. A brief comparison of our new model with the original, constitutive MMTV-PyV mT strain emphasizes the greater experimental practicality of the former (Table 1). In addition to the inherent improvements to the mouse model design (that is, inducibility of the oncogene combined with coupling to Cre recombinase), tumour initiation begins just days after induction of the MIC transgene with complete penetrance to all mammary glands. Importantly, the rtTA/MIC model maintains key desirable traits of its predecessor in terms of tumour progression, in particular, the invasive, metastatic nature of PyV mT mammary tumour cells to distal sites such as the lungs. Perhaps the most useful feature of the MIC strain is that it can be used to study the effect of Cre recombinase-dependent genetic alterations on PyV mT-mediated transformation in any tissue with the appropriate rtTA, making this an important clinical tool for studying many types of cancer in the mouse.

**Table 1:** Comparison of features between the constitutive MMTV-PyV mT/MMTV-Cre recombinase model and the inducible MMTV-rtTA/TetO-MIC model

	**MMTV-PyV mT/MMTV-Cre recombinase**	**MMTV-rtTA/TetO-MIC**
		**(rtTA/MIC [+Dox])**
**PyV mT transgene promoter**	Mouse mammary tumour virus (MMTV)	Tetracycline operator (TetO)
**Inducible transgene expression**	No	Yes
**Coupling to Cre recombinase**	No	Yes
**Time when 50% of animals have tumours (T**_**50**_**)**	40 days of age [6]	7 days of induction
**Tumour pathology**	Solid adenocarcinoma [3,4]	Solid adenocarcinoma
**Cre recombinase expression and function in PyV mT tumour cells (by X-gal assay)**	0% when conditional gene is essential for tumourigenesis [5-7]	100%
**Percentage of tumour-bearing mice with lung metastases**	93.3% [6]	100%

## Abbreviations

amg: adjacent mammary gland; Dox: Doxycycline; EGFR: Epidermal growth factor receptor; EMT: Epithelial-mesenchymal transition; GTRosa: Gene trap ROSA 26; H&E: Haematoxylin and eosin; HRP: Horse radish peroxidase; IRES: Internal ribosome entry sequence; mg: mammary gland; MIC: PyV mT-IRES-Cre recombinase; MIN: Mammary epithelial neoplasia; MMTV: Mouse mammary tumour virus; NIC: Neu-IRES-Cre recombinase; PDGFR: Platelet-derived growth factor receptor; PyV mT: Polyomavirus middle T antigen; qRT-PCR: quantitative reverse transcription polymerase chain reaction; RTK: Receptor tyrosine kinase; rtTA: reverse tetracycline transactivator; SNP: Single nucleotide polymorphism; TetO: Tetracycline operator; TetON: Tetracycline “on”; tum: Mammary tumour.

## Competing interests

We have no competing interests to declare.

## Authors’ contributions

TR and JJR contributed equally to the generation and complete characterization of the transgenic animals. HWS generated and screened the transgenic founder lines and assisted with sample preparation and analyses. SHL performed the molecular cloning of the targeting construct. LC provided the MMTV-rtTA strain. WJM conceptualized the study. TR, JJR, HWS and WJM contributed to writing and revising the manuscript; SHL and LC contributed to critical revision of the manuscript. All authors read and approved the final manuscript

## Supplementary Material

Additional file 1: Figure S1 Figure illustrating doxycycline-inducible expression of PyV mT and Cre recombinase in the mammary epithelium of rtTA/MIC mice. Click here for file

Additional file 2: Figure S2 Figure showing mammary glands from rtTA/MIC mice. Click here for file

Additional file 3: Figure S3 Figure showing adjacent mammary glands from end-stage tumour-bearing rtTA/MIC mice. Click here for file

Additional file 4: Figure S4 Figure showing mammary glands from control animals (both induced and un-induced). Click here for file

Additional file 5: Figure S5 Figure showing multiple histopathologies from recurrent tumours and expression of PyV mT transcript. Click here for file

Additional file 6: Figure S6 Figure showing RTK signalling in doxycycline-independent masses in rtTA/MIC mice. Click here for file
