# Supplementary material for: Inducible and coupled expression of the polyomavirus middle T antigen and Cre recombinase in transgenic mice: an in vivo model for synthetic viability in mammary tumour progression
Source: Breast Cancer Res. 2014 Jan 23;16(1):R11. doi: 10.1186/bcr3603 (PMC3978996; doi:10.1186/bcr3603)
Supplement: Additional file 1: Figure S1 — Figure illustrating doxycycline-inducible expression of PyV mT and Cre recombinase in the mammary epithelium of rtTA/MIC mice. [file bcr3603-S1.pdf]

Figure S1

Doxycycline-inducible expression of PyV mT and Cre recombinase in the mammary epithelium of rtTA/MIC mice

Experimental and control animals were generated by crossing the MMTV-rtTA strain to the TetO-MIC strain. The mouse mammary tumour virus long terminal repeat promoter (MMTV-LTR) directs expression of the reverse tetracycline transactivator (rtTA) to the mammary epithelium. Administration of doxycycline (Dox) to the animal allows the rtTA to bind the Tet operator (TetO) and induce co-expression of PyV mT and Cre recombinase due to an internal ribosome entry sequence (IRES) linking the transgenes.

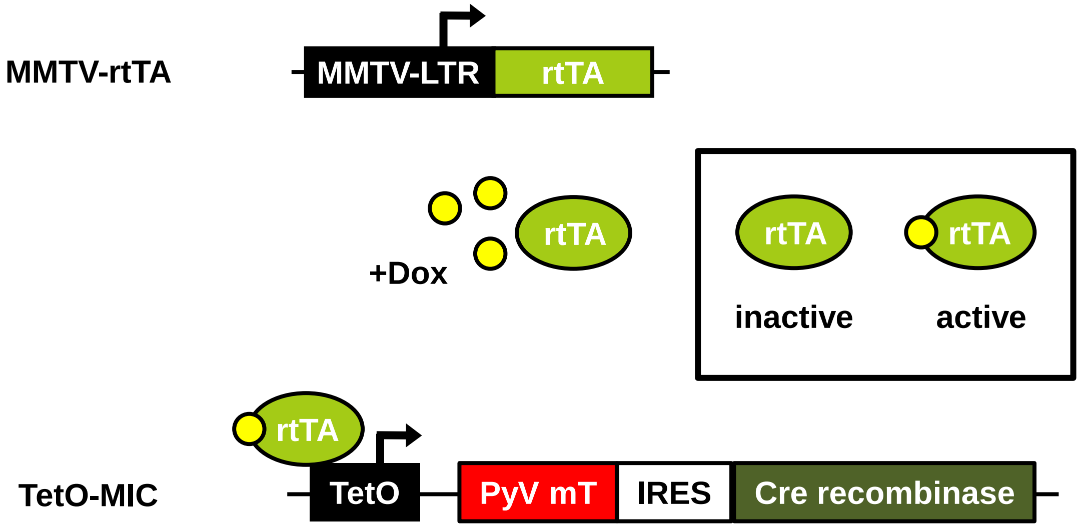

MMTV-rtTA
MMTV-LTR
rtTA
+Dox
rtTA
rtTA
inactive
rtTA
active
rtTA
TetO-MIC
TetO
PyV mT
IRES
Cre recombinase
