# Supplementary material for: Inducible and coupled expression of the polyomavirus middle T antigen and Cre recombinase in transgenic mice: an in vivo model for synthetic viability in mammary tumour progression
Source: Breast Cancer Res. 2014 Jan 23;16(1):R11. doi: 10.1186/bcr3603 (PMC3978996; doi:10.1186/bcr3603)
Supplement: Additional file 2: Figure S2 — Figure showing mammary glands from rtTA/MIC mice. [file bcr3603-S2.pdf]

Figure S2

After 2 weeks of induction, mammary glands from rtTA/MIC mice are transformed

(A) Representative H&E-stained whole mount of a typical rtTA/MIC mammary gland after 2 weeks of induction as compared to a normal mammary gland from an un-induced control. (Scale bars: 0.5mm left; 5mm right).

(B) Representative H&E-stained sections of mammary glands from the same animals shown in (A). (Scale bars: 200µm left; 100µm right).

(C) Quantification of the average number of normal, hyperplastic, and filled ducts relative to the total number of ducts (expressed as a percentage) in mammary gland sections from rtTA/MIC mice induced for 2 weeks and matched controls (induced or un-induced). Error bars represent standard error of mean.

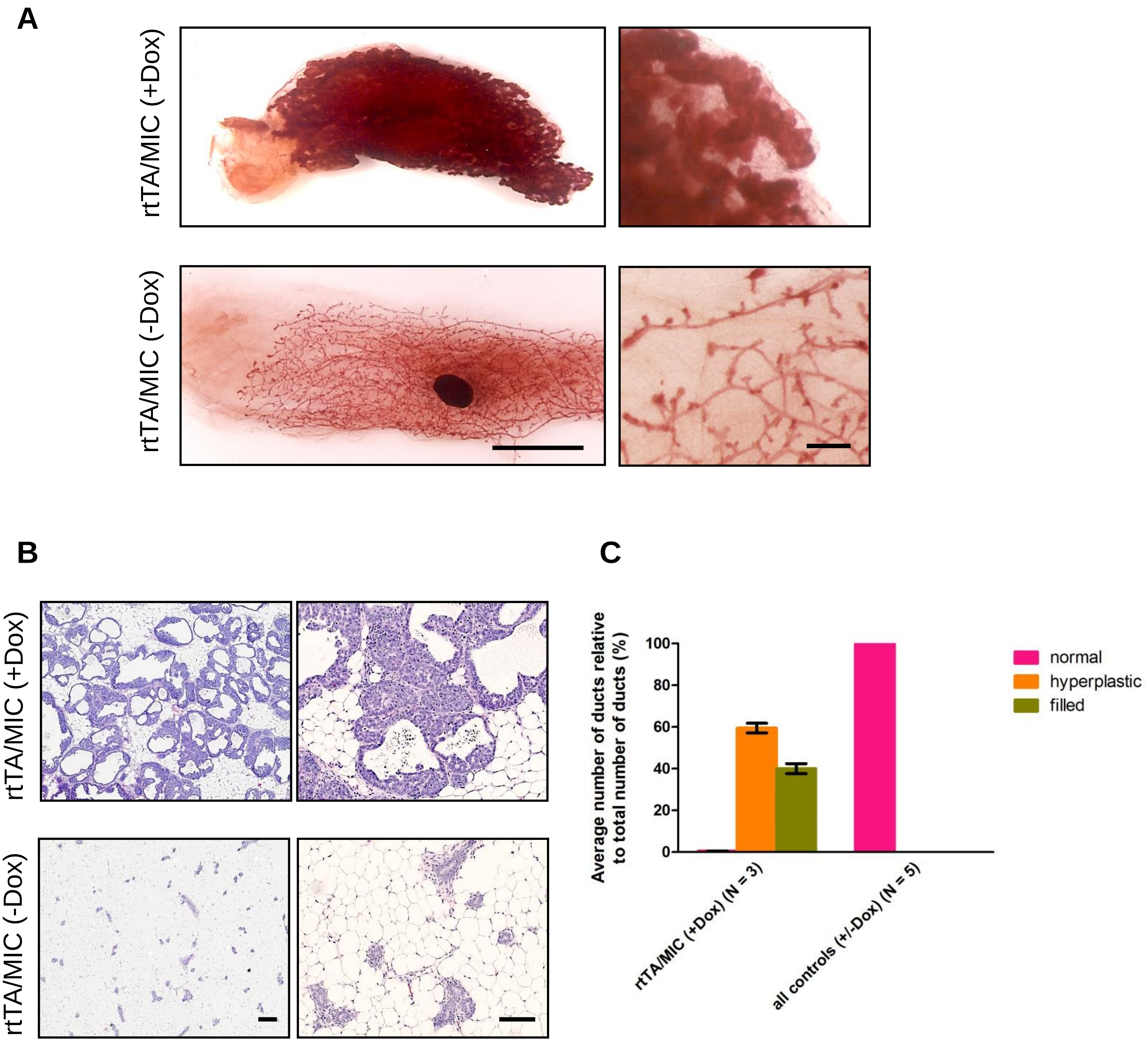

A
rtTA/MIC (+Dox)
rtTA/MIC (-Dox)
B
rtTA/MIC (+Dox)
rtTA/MIC (-Dox)
C
Average number of ducts relative to total number of ducts (%)
100
80
60
40
20
0
rtTA/MIC (+Dox) (N = 3)
all controls (+/-Dox) (N = 5)
normal
hyperplastic
filled
