# Supplementary material for: Inducible and coupled expression of the polyomavirus middle T antigen and Cre recombinase in transgenic mice: an in vivo model for synthetic viability in mammary tumour progression
Source: Breast Cancer Res. 2014 Jan 23;16(1):R11. doi: 10.1186/bcr3603 (PMC3978996; doi:10.1186/bcr3603)
Supplement: Additional file 3: Figure S3 — Figure showing adjacent mammary glands from end-stage tumour-bearing rtTA/MIC mice. [file bcr3603-S3.pdf]

Figure S3

End-stage tumour-bearing rtTA/MIC mice have adjacent mammary glands that are extensively transformed

(A) Representative H&E-stained whole mount preparations of inguinal adjacent mammary glands from rtTA/MIC tumour-bearing mice induced with doxycycline for 9 to 11 weeks. A representative control mammary gland is shown for comparison (an un-induced rtTA/MIC). (Scale bars: 0.5mm left; 5mm, right).

(B) Representative H&E-stained adjacent mammary gland sections from rtTA/MIC tumour-bearing mice induced with doxycycline for 9 to 11 weeks. A representative control mammary gland is shown for comparison (an un-induced rtTA/MIC). Sample genotypes are as indicated in (A). (Scale bars: 0.5mm, left; 2.5mm, right).

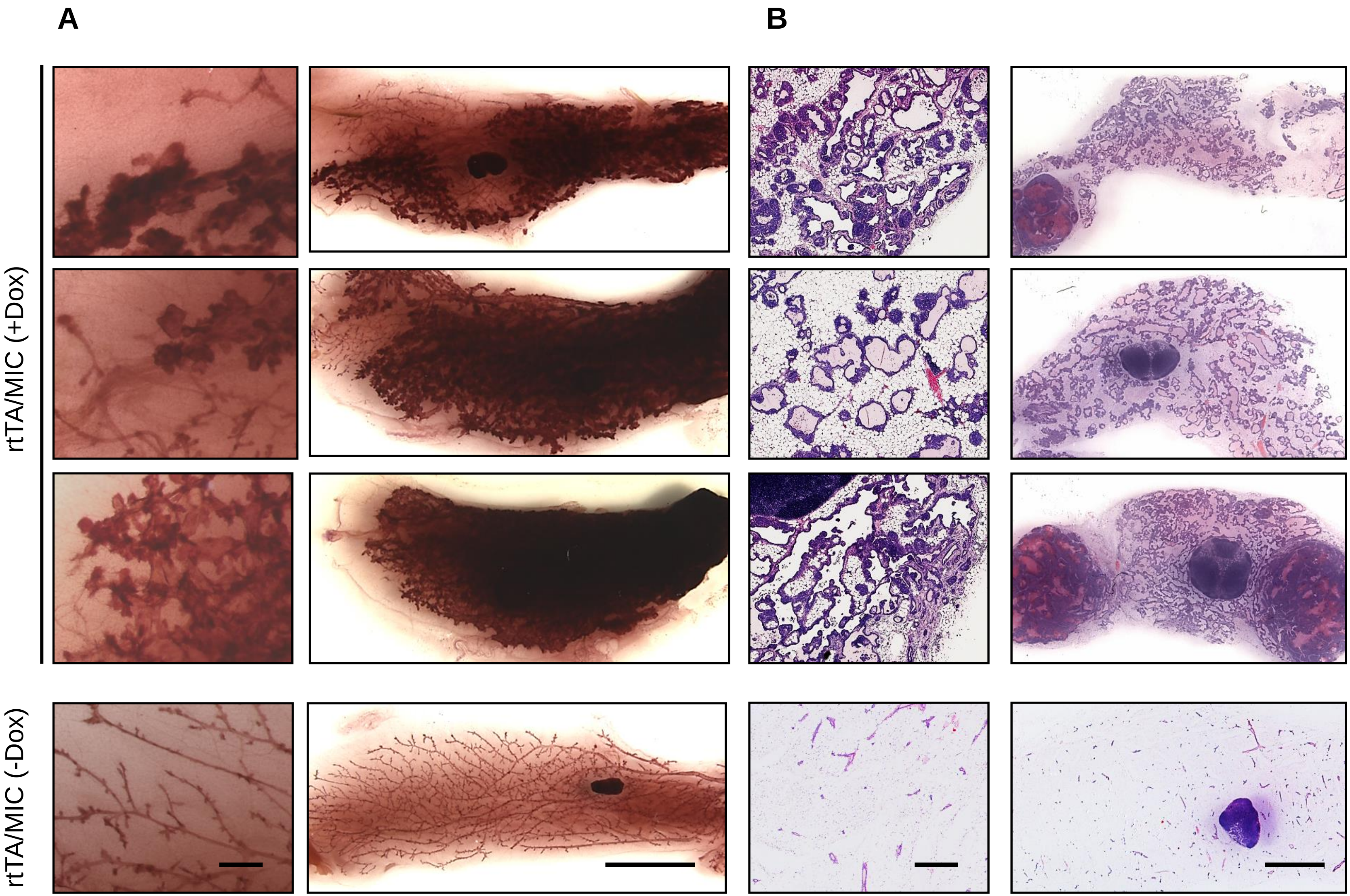
A
B
rtTA/MIC (+Dox)
rtTA/MIC (-Dox)
