# Supplementary material for: Inducible and coupled expression of the polyomavirus middle T antigen and Cre recombinase in transgenic mice: an in vivo model for synthetic viability in mammary tumour progression
Source: Breast Cancer Res. 2014 Jan 23;16(1):R11. doi: 10.1186/bcr3603 (PMC3978996; doi:10.1186/bcr3603)
Supplement: Additional file 4: Figure S4 — Figure showing mammary glands from control animals (both induced and un-induced). [file bcr3603-S4.pdf]

Figure S4

Mammary glands from control animals (both induced and un-induced) are normal

(A) Representative H&E-stained whole mount preparations of inguinal mammary glands from control animals of the indicated genotypes induced for 9 to 11 weeks or left un-induced. (Scale bars: 0.5mm left; 5mm, right).

(B) Representative H&E-staining of mammary gland sections from induced and un-induced control animals of the same genotypes as indicated in (A). (Scale bars: 500µm, left column; 100µm, right column).

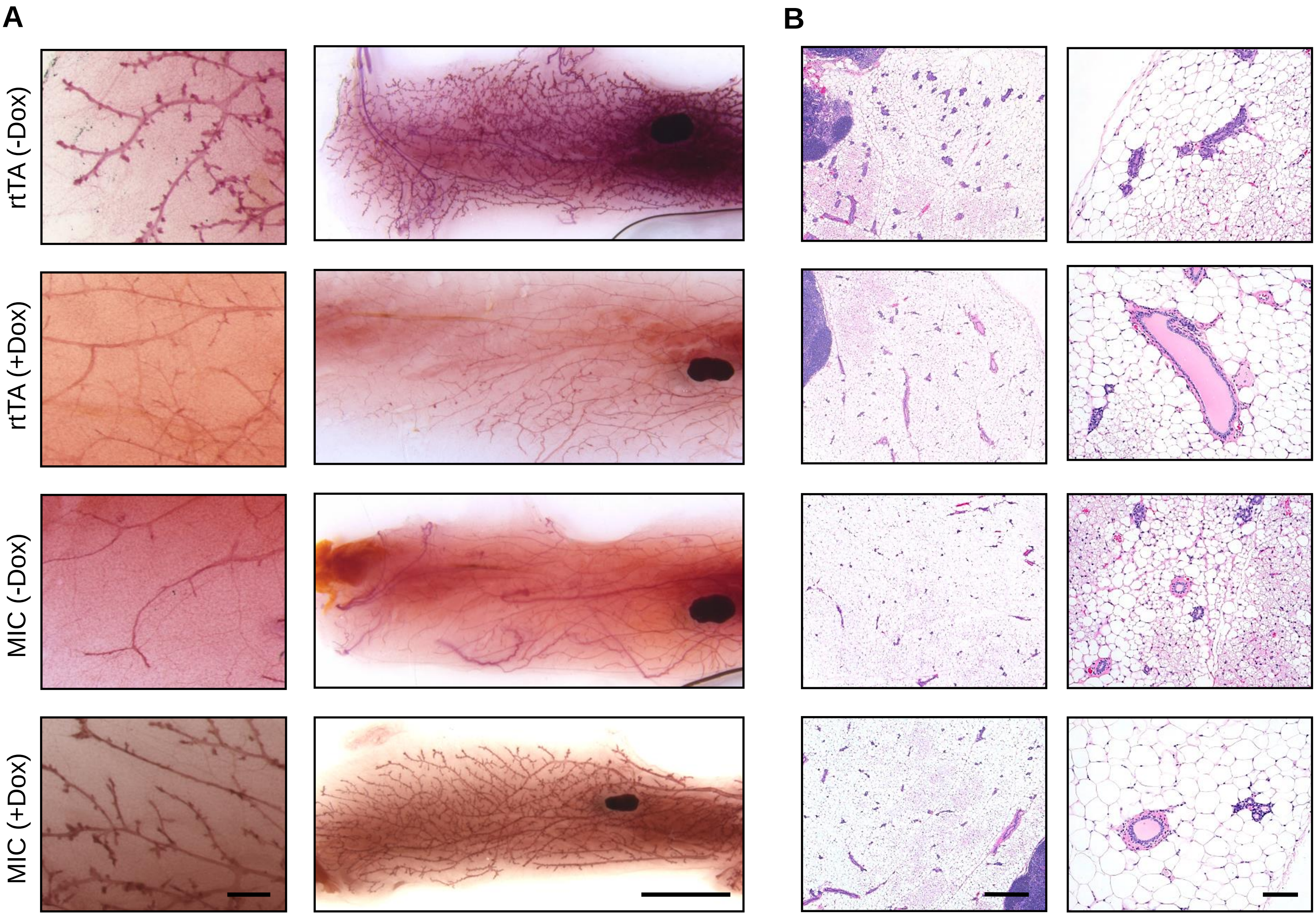
A
B
rtTA (-Dox)
rtTA (+Dox)
MIC (-Dox)
MIC (+Dox)
