# Supplementary material for: Inducible and coupled expression of the polyomavirus middle T antigen and Cre recombinase in transgenic mice: an in vivo model for synthetic viability in mammary tumour progression
Source: Breast Cancer Res. 2014 Jan 23;16(1):R11. doi: 10.1186/bcr3603 (PMC3978996; doi:10.1186/bcr3603)
Supplement: Additional file 5: Figure S5 — Figure showing multiple histopathologies from recurrent tumours and expression of PyV mT transcript. [file bcr3603-S5.pdf]

Figure S5

Multiple histopathologies are observed in recurrent tumours and show variability in re-expression of PyV mT transcript

(A) Select examples of H&E-stained recurrent histopathologies from rtTA/MIC mice post-doxycycline withdrawal. The mouse ID number (followed by the tumour location in the case of multiple recurrences, e.g. “R1”) and histopathology of the tumour are indicated for each image. (Scale bar: 100µm).

(B) PyV mT transcript level as assessed by qPCR on cDNA from primary (rtTA/MIC +Dox) and recurrent (rtTA/MIC +Dox→-Dox) mammary tumours or lung lesions (met). Multiple recurrences in the same animal are indicated by tumour location, e.g. “R1”. Data were normalized to GAPDH transcript. Positive and negative controls were mammary tumour cDNA from MMTV-PyV mT (PyV mT) and MMTV-NIC (NIC) mice, respectively. Error bars represent technical replicates (n=3 for each sample). The incidence of adenocarcinoma in the corresponding histological section for each sample is indicated by a “+” symbol.

A

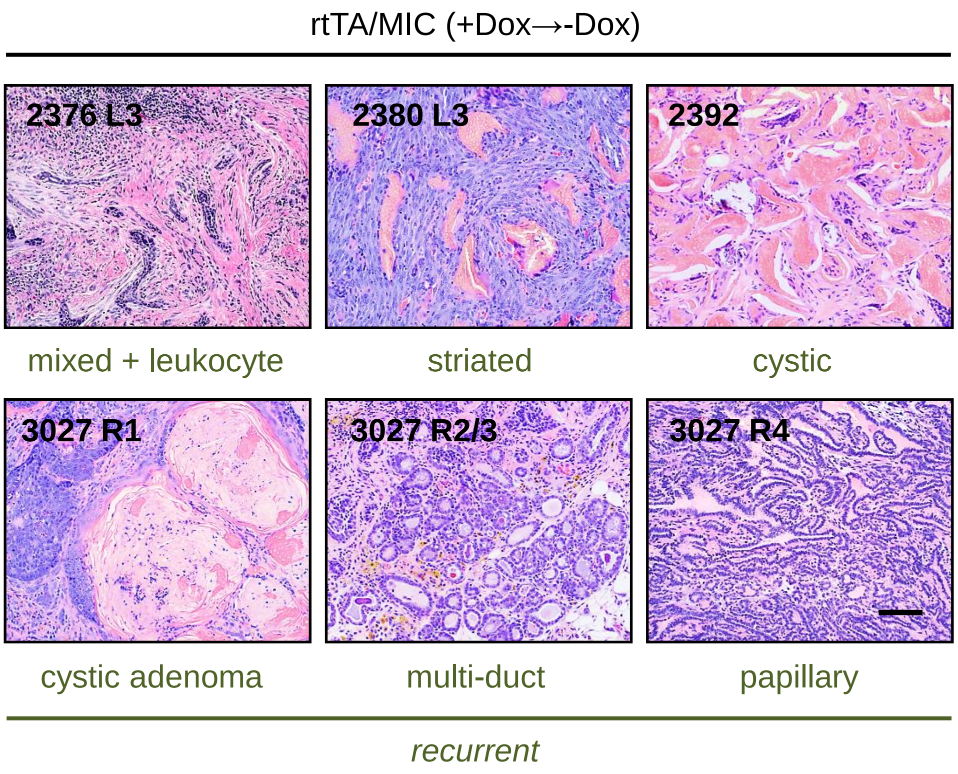
rtTA/MIC (+Dox→-Dox)
2376 L3
2380 L3
2392
mixed + leukocyte
striated
cystic
3027 R1
3027 R2/3
3027 R4
cystic adenoma
multi-duct
papillary
recurrent

B

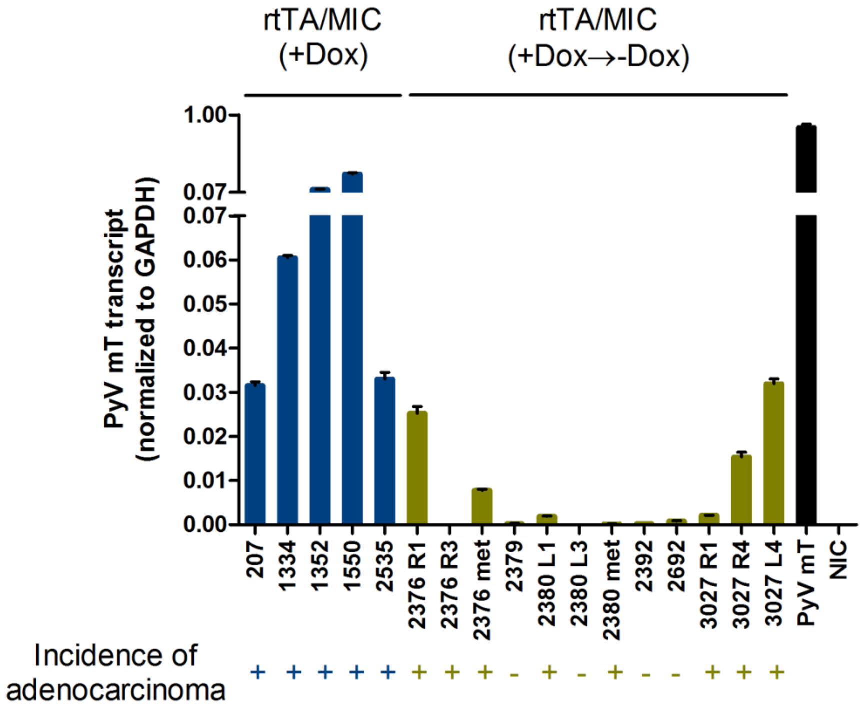
rtTA/MIC
(+Dox)
rtTA/MIC
(+Dox→-Dox)
PyV mT transcript
(normalized to GAPDH)
1.00
0.07
0.07
0.06
0.05
0.04
0.03
0.02
0.01
0.00
207
1334
1352
1550
2535
2376 R1
2376 R3
2376 met
2379
2380 L1
2380 L3
2380 met
2392
2692
3027 R1
3027 R4
3027 L4
PyV mT
NIC
Incidence of
adenocarcinoma
+ + + + + + + + - + - + - - + + +
