# Supplementary material for: Inducible and coupled expression of the polyomavirus middle T antigen and Cre recombinase in transgenic mice: an in vivo model for synthetic viability in mammary tumour progression
Source: Breast Cancer Res. 2014 Jan 23;16(1):R11. doi: 10.1186/bcr3603 (PMC3978996; doi:10.1186/bcr3603)
Supplement: Additional file 6: Figure S6 — Figure showing RTK signalling in doxycycline-independent masses in rtTA/MIC mice. [file bcr3603-S6.pdf]

Figure S6

Doxycycline-independent masses in rtTA/MIC mice display RTK signalling that may represent mechanisms of recurrence

(A) Levels of phosphorylated EGFR, ErbB2, and PDGFRβ in primary and recurrent rtTA/MIC mammary tumours expressed as fluorescence intensity of the probe on the array normalized to PBS (negative control probe). An MMTV-PyV mT (PyV mT) mammary tumour was used as a control.

(B) Immunoblot analysis of protein lysates from rtTA/MIC mammary tumours prior to and following doxycycline withdrawal using antibodies directed to P-EGFR (Y1068), EGFR, P-ErbB2 (Y1248), ErbB2, P-PDGFRβ (Y1021), PDGFRβ, c-Myc and Hsp90 (loading control). Resected mammary tumours were used for pre- and mid-regression time-points (pre and mid, respectively), while recurrent masses were harvested from mice sacrificed at clinical endpoint. The incidence of adenocarcinoma in the corresponding histological section for each sample is indicated by a "+" symbol.

A

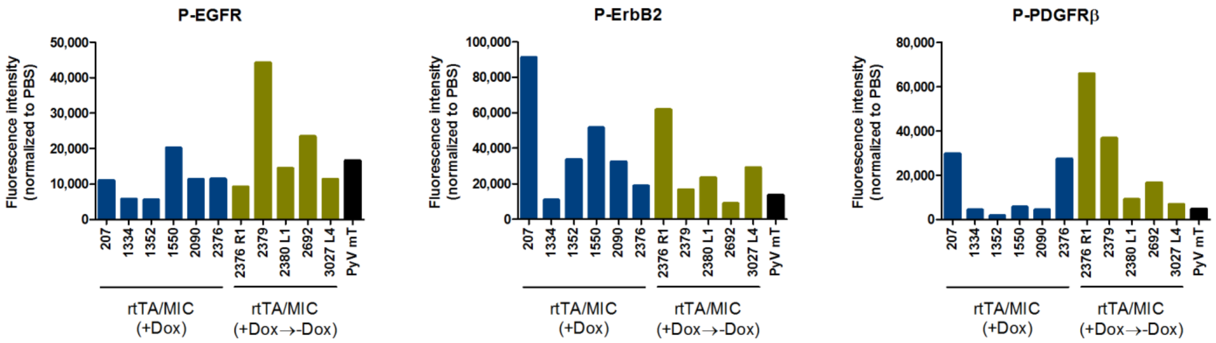

P-EGFR
P-ErbB2
P-PDGFRβ
Fluorescence intensity (normalized to PBS)
207
1334
1352
1550
2090
2376
2376 R1
2379
2380 L1
2692
3027 L4
PyV mT
rtTA/MIC (+Dox)
rtTA/MIC (+Dox→-Dox)

B

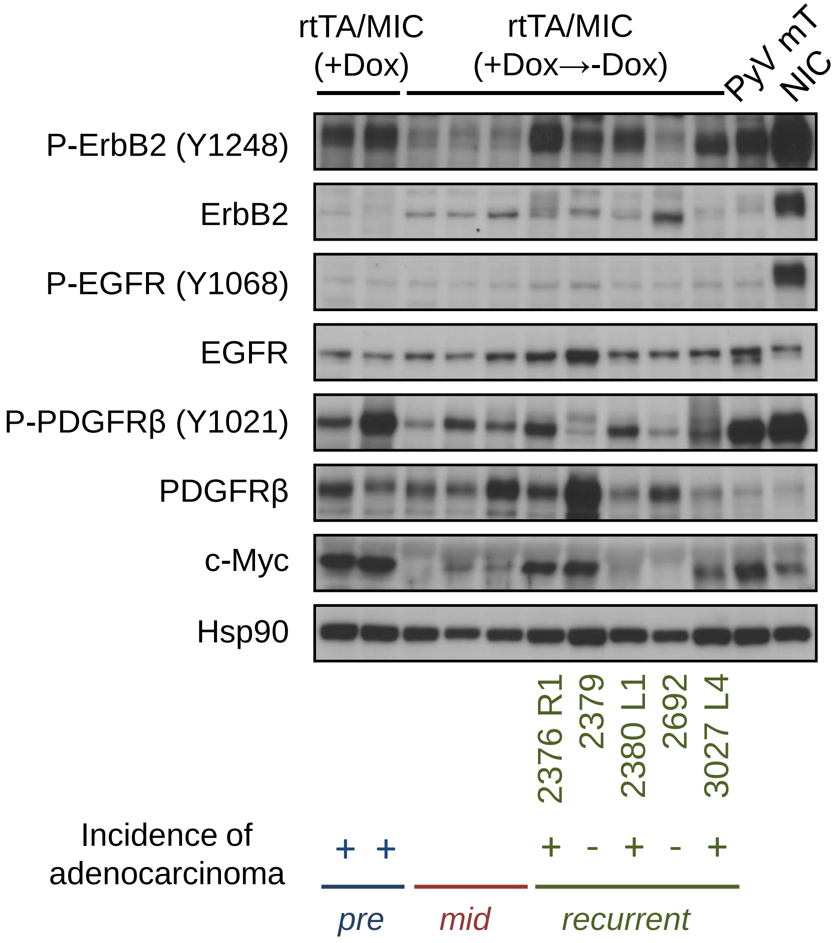

rtTA/MIC (+Dox)
rtTA/MIC (+Dox→-Dox)
PyV mT
NIC
P-ErbB2 (Y1248)
ErbB2
P-EGFR (Y1068)
EGFR
P-PDGFRβ (Y1021)
PDGFRβ
c-Myc
Hsp90
2376 R1
2379
2380 L1
2692
3027 L4
Incidence of adenocarcinoma
+ +
+ - + - +
pre
mid
recurrent
